# Safety Considerations of Adaptive Horseback Riding Program for Adults With Dementia and Their Families

**DOI:** 10.1093/geroni/igab046.695

**Published:** 2021-12-17

**Authors:** Alicia Oestreich, Beth Fields

**Affiliations:** University of Wisconsin-Madison, Madison, Wisconsin, United States

## Abstract

Human-animal interactions, including equine-assisted services, are becoming increasingly popular to enhance the quality of life of adults with dementia and their families. However, there is a lack of knowledge on safety considerations when serving this population. The purpose of this qualitative descriptive study was to explore the safety perspectives of key stakeholders involved in an adaptive horseback riding program for adults with dementia and their families. Ten, 30-minute semi-structured interviews and two, 60-minute focus groups were conducted with horseback riding program instructors and staff, dementia specialists, and adults with dementia and their families. Thematic analysis of data were guided by the Professional Association of Therapeutic Horsemanship International’s Core Safety Standards and completed using NVivo 12. Stakeholders described two central themes to consider when offering equine-assisted services at therapeutic horseback riding centers to adults with dementia and their families 1) dementia and horse training parameters, and 2) enrollment procedures. Stakeholders recommended that training should encompass “how to interact and communicate in a positive way with the adult with dementia…redirect if a behavior comes up”, and horses should be specially selected to “tolerate standing in a ramp during a difficult mount”. Stakeholders also shared that enrollment in the program should include learning the adult’s health and prior horse experience, precautions and contraindications, horseback riding readiness, and availability of and support from family. Information gleaned from this study may help researchers, instructors, and staff develop policies that demonstrate optimal safety practices when delivering equine-assisted services to adults with dementia and their families.

